# Intricate tunnels in garnets from soils and river sediments in Thailand – Possible endolithic microborings

**DOI:** 10.1371/journal.pone.0200351

**Published:** 2018-08-08

**Authors:** Magnus Ivarsson, Henrik Skogby, Bongkot Phichaikamjornwut, Stefan Bengtson, Sandra Siljeström, Prayote Ounchanum, Apichet Boonsoong, Mingkhwan Kruachanta, Federica Marone, Veneta Belivanova, Sara Holmström

**Affiliations:** 1 University of Southern Denmark, Department of Biology and Nordic Center for Earth Evolution, Odense M, Denmark; 2 Swedish Museum of Natural History, Department of Palaeobiology, Stockholm, Sweden; 3 Swedish Museum of Natural History, Department of Geosciences, Stockholm, Sweden; 4 Gems and Jewelry Program, Faculty of Science, Srinakharinwirot University, Bangkok, Thailand; 5 RISE Research Institutes of Sweden, Bioscience and Materials/Chemistry and Materials, Stockholm, Sweden; 6 Department of Geological Sciences, Faculty of Science, Chiang Mai University, Chiang Mai, Thailand; 7 Swiss Light Source, Paul Scherrer Institute, Villigen, Switzerland; 8 Stockholm University, Department of Geological Sciences, Stockholm, Sweden; Niagara University, UNITED STATES

## Abstract

Garnets from disparate geographical environments and origins such as oxidized soils and river sediments in Thailand host intricate systems of microsized tunnels that significantly decrease the quality and value of the garnets as gems. The origin of such tunneling has previously been attributed to abiotic processes. Here we present physical and chemical remains of endolithic microorganisms within the tunnels and discuss a probable biological origin of the tunnels. Extensive investigations with synchrotron-radiation X-ray tomographic microscopy (SRXTM) reveal morphological indications of biogenicity that further support a euendolithic interpretation. We suggest that the production of the tunnels was initiated by a combination of abiotic and biological processes, and that at later stages biological processes came to dominate. In environments such as river sediments and oxidized soils garnets are among the few remaining sources of bio-available Fe^2+^, thus it is likely that microbially mediated boring of the garnets has trophic reasons. Whatever the reason for garnet boring, the tunnel system represents a new endolithic habitat in a hard silicate mineral otherwise known to be resistant to abrasion and chemical attack.

## Introduction

Endoliths are microorganisms living inside substrates, mostly rocks and minerals, but also shells, corals or wood [1,2]. Endolithic lineages have been developed among bacteria, fungi, algae, and several animal phyla, and they can either be chemolithoautotrophs (which utilize inorganically stored energy and carbon from inorganic sources like minerals), heterotrophs, or even photoautotrophs (like cyanobacteria) [2,3]. The usual advantage of entertaining an endolithic lifestyle is to obtain residence space—a hard or soft substrate provides a stable and protected environment compared to the outside. However, heterotrophs and chemolithoautotrophs may bore a substrate for trophic reasons as well. Saprophytic fungi, for instance, frequently bore into wood and bone [3], and mycorrhizal fungi are known to bore into soil minerals to mobilize nutrients for symbiotic plants [4,5]. Prokaryotic microborers are believed to bore in volcanic glass to oxidize reduced iron and manganese species for their metabolism [6,7].

Endoliths are assigned to subcategories depending on their way of life and occurrence within the substrate [1]: euendoliths actively penetrate the substrate to produce residential cavities, cryptoendoliths inhabit pre-existent structural cavities, and chasmoendoliths colonize pre-existent cracks and fissures. A fourth type; autoendoliths, has recently been distinguished and is defined as organisms that construct the structures in which they reside through pore space filling [8].

Euendolithic activity leaves various types of etch marks in the penetrated substrate. Such etch marks can range from surficial irregular cavities to complex tunnel structures that reach deep within a substrate [6,7]. The shape of the produced cavity is to a large extent controlled by the physiology of the boring microorganism. Coccoidal cells usually produce shallow etch marks [9,10] while filamentous organisms produce long tunnel-like structures, either horizontal on a mineral surface or deep in a substrate at varying angles to the surface [10,11]. Complex bioeroded tunnel structures are more demanding to produce than shallow etch marks, and they are considered as remains of more complex organisms like fungi, algae or certain types of filamentous bacteria like cyanobacteria [2]. The physical marks of euendoliths are classified as trace fossils or ichnofossils, and they can be used in retrospect to understand what organisms were responsible, and why and how the organisms produced the cavity [12]. However, as always with microfossils, one needs to be aware of abiotic alternatives. There are ways to produce both etch marks and tunnels abiotically, and this needs to be taken into account when studying ichnofossils [13].

The residential cavities of euendoliths are usually explained as the result of either physical force but more likely through chemical dissolution [6,7]. A plethora of microorganisms including bacteria, fungi and algae are known to chemically etch minerals by excreting organic acids or chelators, such as siderophores, that act corrosively to certain minerals or elements [14,15]. Minerals react differently to acid attack; carbonates and phosphates for example react relatively easy to acid attacks while other minerals, like silicates [16], react slower or not at all to acids, and are thus harder to dissolve and penetrate. Usually, minerals with hardness from seven and above on the Mohs scale are to our knowledge seldom bored, and have so far not been associated with euendolithic borings. Minerals and materials with high redox potential, like volcanic glass, are also relatively frequently corroded [4]. However, in a highly oxidized environment, where accessible elements or easily penetrated substrates are used and thus rare, the microorganisms need to adapt and invent strategies to penetrate less attractive and easily dissolved substrates to access bioavailable elements.

Phichaikamjornwut *et al*. [17] described complex tunnel-like networks in garnets of gem quality from Thailand as the result of abiotic processes, but discussed briefly a possible biology-assisted leaching process being involved as well. The tubular structures significantly decrease the quality and value of the garnets as gems. A confirmation of microorganisms actively dissolving and boring into such a hard mineral as garnet would force reconsiderations upon the capability of microorganisms as microborers but also the adaptability of microorganisms in low-nutrient environments such as river sediments and oxidized soils. Cavities in garnets have been shown to contain carbonaceous material of biological origin from deep ultramafic rocks indicating the presence of deep subseafloor life [18]. Inclusion trails in garnets from Eoarchean metasedimentary rocks from Isua, West Greenland, also contain carbonaceous material, which represents the oldest biogenic carbon relics on Earth [19]. The growing awareness of garnets as minerals in which biogenic carbon is being preserved calls for a deeper understanding of the mechanisms behind the formation of the cavities and the possible involvement of endolithic microorganisms.

Here we report endolithic remains in garnets (pyrope and almandine) from river sediments and soils in Thailand. Garnets are relatively hard minerals (H_pyrope_ = 7.5) resistant to abrasion and chemical attack, and have not previously been identified as endolithic habitats. The endolith remains are observed in an intricate tunnel system characterized by frequent branching and anastomoses between branches, bearing close resemblance to biological features. We discuss the presence of biomarkers and microbial-like structures within these tunnels and a possible biological involvement in their production.

## Methods

### Geological settings

The samples in the current study are from three types of geological settings in northern- and mid-Thailand, (1) residual soils from in-situ weathered basalt: Khao Wua, Chantaburi Province (KW) (12°37'23"N,102°3'6"E), Bo Rai, Trat Province (BR) (12°34'18"N,102°31'6"E), Nong Bon, Trat Province (NB) (12°40'30"N,102°27'52"E), (2) granites: Ob Luang (OL) (18°22'47"N,98°31'57"E), Chom Thong District, and (3) river sediments: Chiang Mai stream, Chom Thong, Chiang Mai Province (CT) (18°24'5"N,98°38'18"E) (see fig 3.1 in [20] for map of the sample areas). Hereafter only the abbreviations will be used. No permits were required for the described study, which complied with all relevant regulations.

The Chantaburi area has been covered by Quaternary basalts dated at 0.44±0.11 Ma [21]. The basalts are generally strongly alkaline, with a low silica and high titanium content. They are fine-grained, olivine-bearing, and occasionally contain clinopyroxene and chromium-rich spinel megacrysts with mantle-derived spinel lherzolite xenoliths. The garnets are found in the residual soils of the in-situ mass-scale weathering of the basalts.

The Trat Province is characterized by sedimentary and metamorphic rocks of Permian-Carboniferous age including siltstone, mudstone, tuffaceous sandstone, agglomerate, and locally interbedded conglomerate lenses. These rocks are overlain by basalts classified as nephelinite and olivine nephelinite of Triassic age, which are the source rocks of the studied garnets.

Ob Luang (OL), Chom Thong District, Chiang Mai Province lies within a “Chiang Mai-Tak Gneiss Belt” which is a part of the so-called “Crystalline Basement” or “Basement Complex”, a structurally complex igneous and metamorphic formation of presumed Precambrian age.

Chiang Mai stream, Chom Thong, Chiang Mai Province (CT), contain river sediments that have been transported from OL. The investigated areas are underlain by Precambrian metamorphic complexes of amphibolites facies or anatexitic aureole with relics of Precambrian paragneisses. The rocks consist of anatexite or migmatite, augen gneiss, marble, calc-silicate rocks and quartz-mica schist [22–26].

### Sample preparation

The samples were initially investigated under optical light microscope, environmental scanning electron microscope (ESEM) and scanning electron microscope (SEM). For optical microscopy the samples were cut and polished in two-faced sections. For ESEM analysis garnets were embedded in epoxy and polished. To characterize the galleries of tunnels in the garnets synchrotron-radiation X-ray tomographic microscopy (SRXTM) was used. To detect and characterize organic compounds in the tunnels time-of-flight secondary ion mass spectrometry (ToF-SIMS) was used.

A stereozoom microscope (up to 75-X magnification) coupled with several illumination techniques was used to study the garnets.

### ESEM

For ESEM analyses an XL30 microscope with a field emission gun (XL30 ESEM-FEG) was used equipped with an Oxford x-act energy dispersive spectrometer (EDS), backscatter electron detector (BSE), and a secondary electron detector (SE). The acceleration voltage was 20 or 15 kV depending on the nature of the sample. The instrument was calibrated with a cobalt standard. Peak and element analyses were made using INCA Suite 4.11 software.

SEM images of garnets, which had previously been analysed by ToF-SIMS, were also acquired using a Supra 40 VP FEG SEM (Zeiss, Germany) at RISE Research Institutes of Sweden operating at 2 keV in secondary electron mode. The garnets were gold-coated before the SEM analyses.

### SRXTM

SRXTM was carried out at the TOMCAT beamline of the Swiss Light Source, Paul Scherrer Institute, Villigen, Switzerland. X-ray energies employed varied from 15 to 35 keV, allowing for optimal penetration. A total of 1501 projections were acquired during rotation of the specimen over 180°, post-processed and rearranged into flat- and darkfield-corrected sinograms. Reconstruction was performed on a Linux PC farm using highly optimized routines based on the Fourier Transform method [27,28]. Slice data derived from the scans were then rendered using Avizo® software. Lenses used were x10 and x20, resulting in a voxel size of 0.74 μm and 0.37 μm, respectively.

### ToF-SIMS

For ToF-SIMS analyses garnets with high degree of tunnelling were selected from the KW and CT area. They were kept in aluminium foil, treated with stainless steel forceps and cracked under sterile conditions to avoid contamination. The garnets were split in a laminar flow hood right before analyses using a cleaned chisel (heptane, acetone and ethanol in that order). They were then mounted with clean tweezers on double-sticky tape on a silica wafer. As negative controls, other minerals (quartz and hematite) collected in the same samples as the garnets, were analysed with ToF-SIMS. Analyses were performed both on the outside surfaces of the minerals and newly exposed surfaces (split with a clean chisel).

The ToF-SIMS analysis was performed on a ToF-SIMS IV (ION-TOF GmbH) by rastering a 25 keV Bi_3_^+^ beam (pulsed current of 0.1 pA) over an area of ~200x200 μm for 200–300 sec. The analyses were performed in positive and negative mode at high mass resolution (bunched mode: Δl ~ 3 μm, m⁄Δm ~ 2000–4000 at m⁄z 30). As a control, additional spectra were also acquired from the tape to confirm that samples had not been contaminated by the tape.

## Results

### Mineralogy

The garnets in the current study are pyrope—almandine within the compositional ranges Py_59-68_Al_18-26_Gr_11-13_ and Al_70-75_Sp_9-22_Py_8-14_. Most garnets contain hollow or filled tunnel-like structures except the OL garnets, which were sampled from granite close to the Chiang Mai stream. The reason for sampling OL garnets is that they are considered to be the source for the CT garnets sampled downstream in a tributary to the Chiang Mai River. Thus, the CT garnets likely represent the weathering product of the host rock in OL. The garnets from the host rock (OL) are homogenous with no visible inclusions or tunnel structures. The garnets sampled downstream (CT) have a high degree of tunneling.

### Tunnels

**Fig 1:**
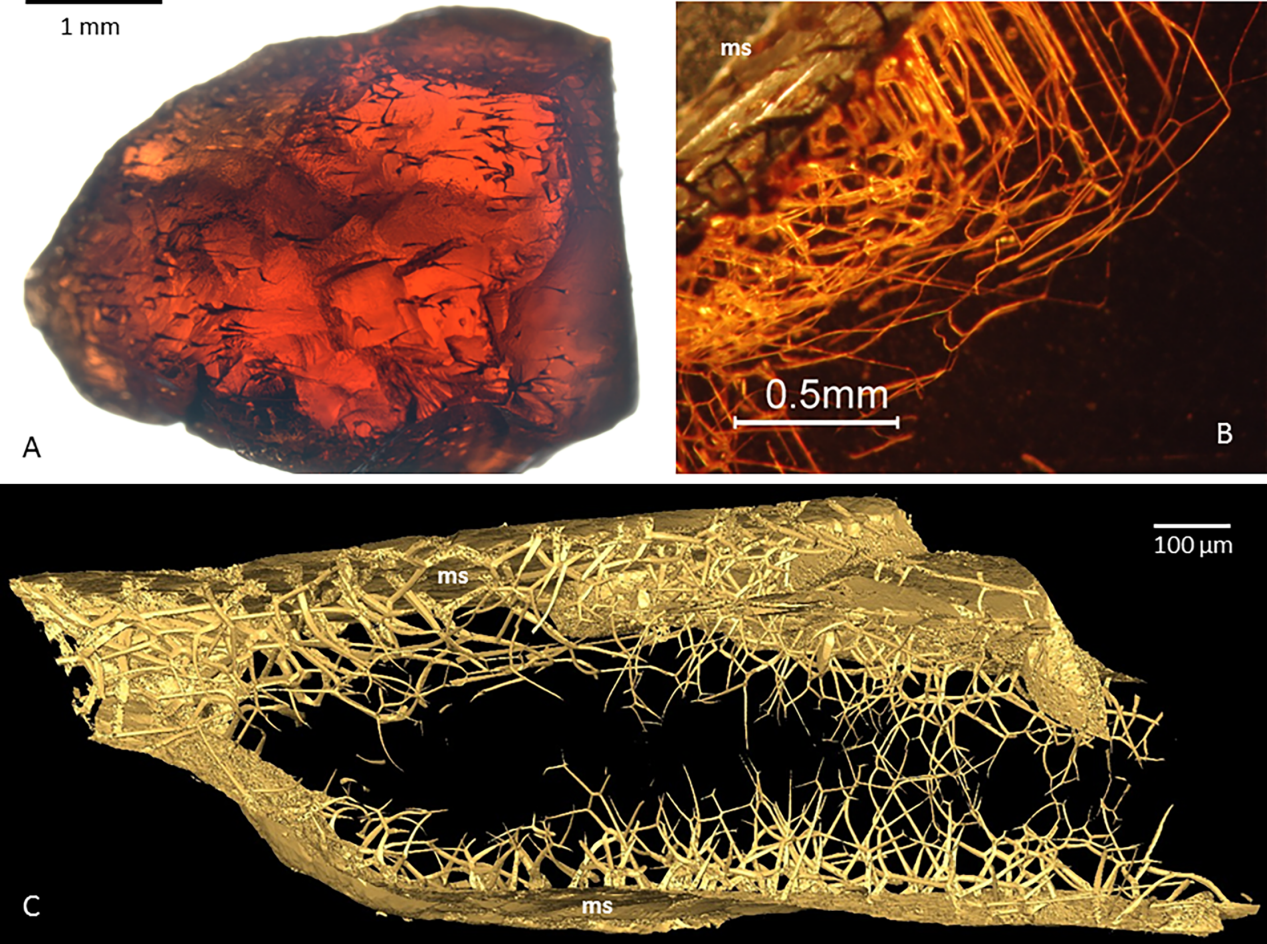
NB pyropes. A) Photograph of a garnet crystal with distinct tubular structures. B) Microphotograph of network of tubular structures originating at the mineral surface and stretching into the garnet relatively localized to the margin of the garnet. C) Tomographic reconstruction (isosurface rendering) of a garnet crystal with network of tubular structures originating at the mineral surface and stretching inwards into the crystal interior. The interior of the crystal are made black to make the tubular structures more visible. Legend: ms, mineral surface.

**Fig 2:**
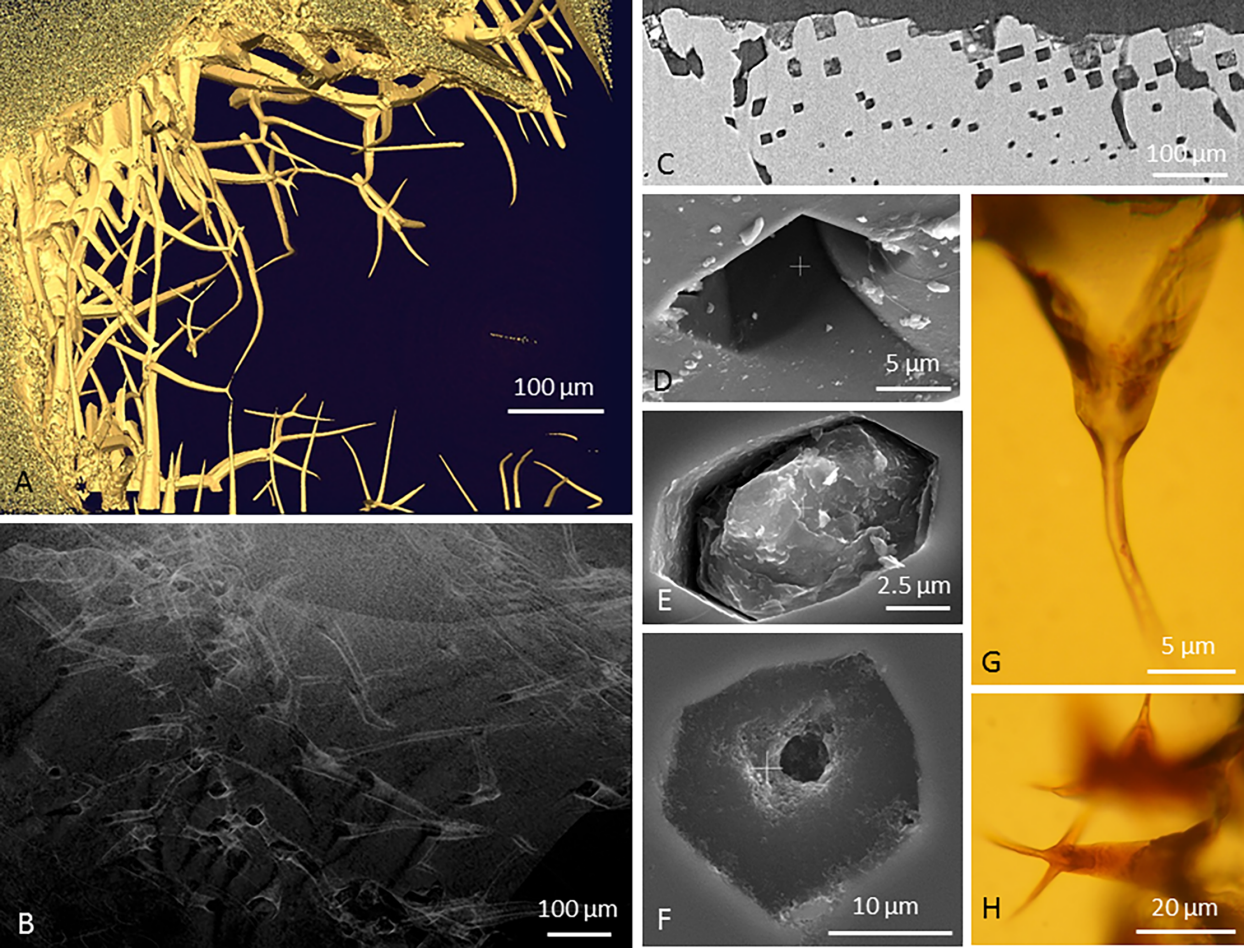
Images A-B: CT almandines; C-F: KW pyropes; G-H: CT pyropes. A) Tomographic reconstruction (isosurface rendering) of a garnet with tubular structures originating at the surface. The bases of the tunnels are broad and have distinct hexagonal or rectangular cross sections but tapering off and become more rounded toward the tips. B) Tomographic reconstruction (volumetric rendering) showing the hexagonal cross section of multiple tubular structures. C) An orthoslice of a tomographic reconstruction showing the cross-sectional hexagons or rectangles of the tunnels. D) SEM image of a four-angled polygonal entrance hole. E) SEM image of a six-angled polygonal entrance hole that is filled. F) SEM image of a tubular structure that tapers off further into the mineral. Note how the tunnel have a polygonal shape at the mineral surface but further in gets more circular as it tapers off. G) Microphotograph of a tubular structure that tapers off and also starts with a polygonal shape at the mineral surface but gets more circular as it penetrates further into the mineral and tapers off. H) Microphotograph of tubular structures that tapers off. The branching of the tunnels results in offspring tunnels with less diameter than the originating tunnel.

The tunnels all originate from the grain surface and extend into the mineral (Fig 1). They are typically funnel-shaped, with hexagonal or rectangular cross sections in the coarser portions (Fig 2A–2F), attaining more rounded shape towards the tips (Fig 2F–2H). The orientation of the cross-sectional hexagons or rectangles is typically identical in adjacent tunnels, suggesting that the shape is controlled by crystal planes (Fig 2C). The diameter at the opening varies considerably; from about 5 μm to about 100 μm. Broader tunnels show the highest degree of narrowing, whereas those starting narrow tend to be more equidimensional along most of their length (Fig 1B and 1C). Tunnels are usually straight near their opening to the mineral surface but tend to change direction or branch toward the tips. The direction may sometimes change sharply with a kink (Fig 1B), but commonly the tunnels follow a smooth and sinuous curvature, suggesting that the direction is not primarily governed by crystallography (Figs 1C and 2A).

**Fig 3:**
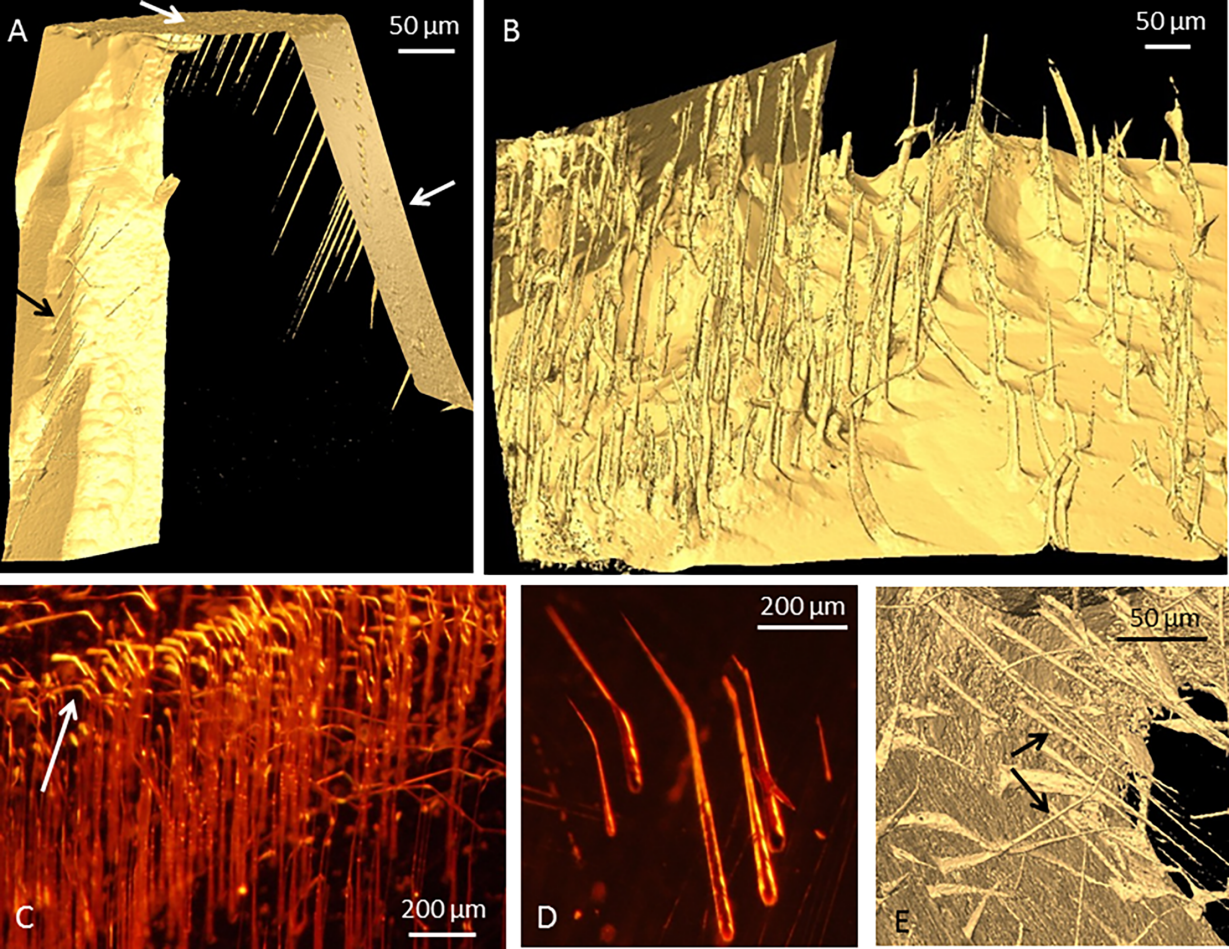
Images A-B: BR pyropes; C-E: NB pyropes. A) Tomographic reconstruction (isosurface rendering) showing straight and strictly parallel tunnels entering from three different mineral surfaces forming almost perfect rows. Arrows mark the three different mineral surfaces. White arrows mark external mineral surfaces and the black arrow marks the internal mineral surface. B) Tomographic reconstruction (isosurface rendering) of tunnels, although parallel, but not lined up and more irregularly scattered. C) Microphotograph of parallel tunnels with a seemingly coordinated curvature of the distal parts of each tunnel. Arrow marks the curvature. D) Microphotograph of a few parallel tunnels with a common curvature. E) Tomographic reconstruction (isosurface rendering) of palisades within a crystal, where internally parallel tunnels in each set make distinctive angles to co-occurring sets projecting in other directions. See arrows.

The tunneling displays a substantial range of appearance and morphological traits, from strictly organized palisades of parallel tunnels to irregularly branching and anastomosing networks. In the most organized variety, straight and strictly parallel tunnels form almost perfect rows (Fig 3A); more commonly the tunnels, although parallel, are not lined up but are more irregularly scattered (Fig 3B). A recurring feature is a parallel, seemingly coordinated, curvature of the distal parts of each tunnel in such palisades (Fig 3C and 3D). There may also be two or more sets of palisades within a crystal, where internally parallel tunnels in each set make distinctive angles to co-occurring sets projecting in other directions (Fig 3E).

**Fig 4:**
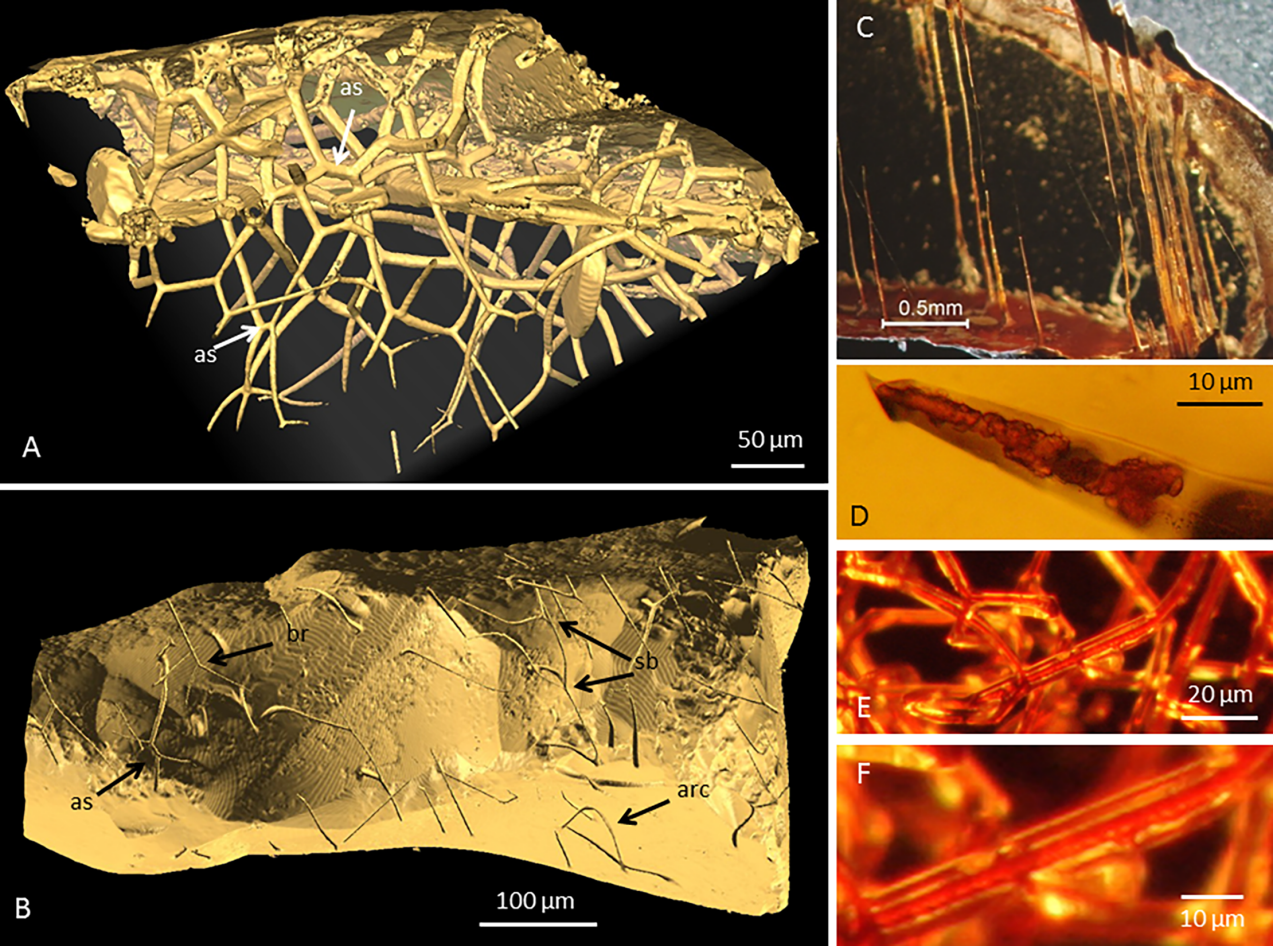
Images of CT pyropes. A) Tomographic reconstruction (isosurface rendering) of a network of tubular structures forming a complex network with frequent branching and anastomoses between branches. B) Tomographic reconstruction (isosurface rendering) of a garnet showing tubular structures originating at the mineral surfaces penetrating into the mineral. The network is characterized by branching but also anastomosis between branches. An arc-shaped tubular structure is also seen. Legend: br, branching; sb, serial branching; as, anastomosis; arc, arc-shaped tubular structure. C) Microphotograph of part of a garnet with straight, parallel tunnels that reach from one side of the garnet to the opposite side. D) Microphotograph of a tubular structure that contains a reddish filament-structure with precipitations on its surfaces. E) Microphotograph of tunnels with a reddish filamentous filling. F) Close-up microphotograph of the filamentous structure inside the tunnel in E.

At the other end of the morphological range are the branching and anastomosing networks that may begin as straight tunnels but thereafter split dichotomously at one or more successive branching points. Frequently a split-off branch joins a neighboring tunnel, so that a more-or-less complex network ensues (Fig 4A and 4B). The same interconnected branching tunnel system can thus have contact with the mineral surface at several sites (Fig 4B). At some branches the side branches are smaller in diameter than the original tunnel, but this is not exclusively the case and the side branches can also have the same diameter as the original tunnels. The branching, serial branching and anastomosing behavior of the tunnels results in intricate and complex networks of tunnels that stretch from the mineral surface and deep into the mineral grains.

Some tunnels are arch-shaped with both ends at the same surface close to each other (Fig 4B). Others reach from one side of a grain to the other, stretching right through the mineral grain (Fig 4C).

**Fig 5:**
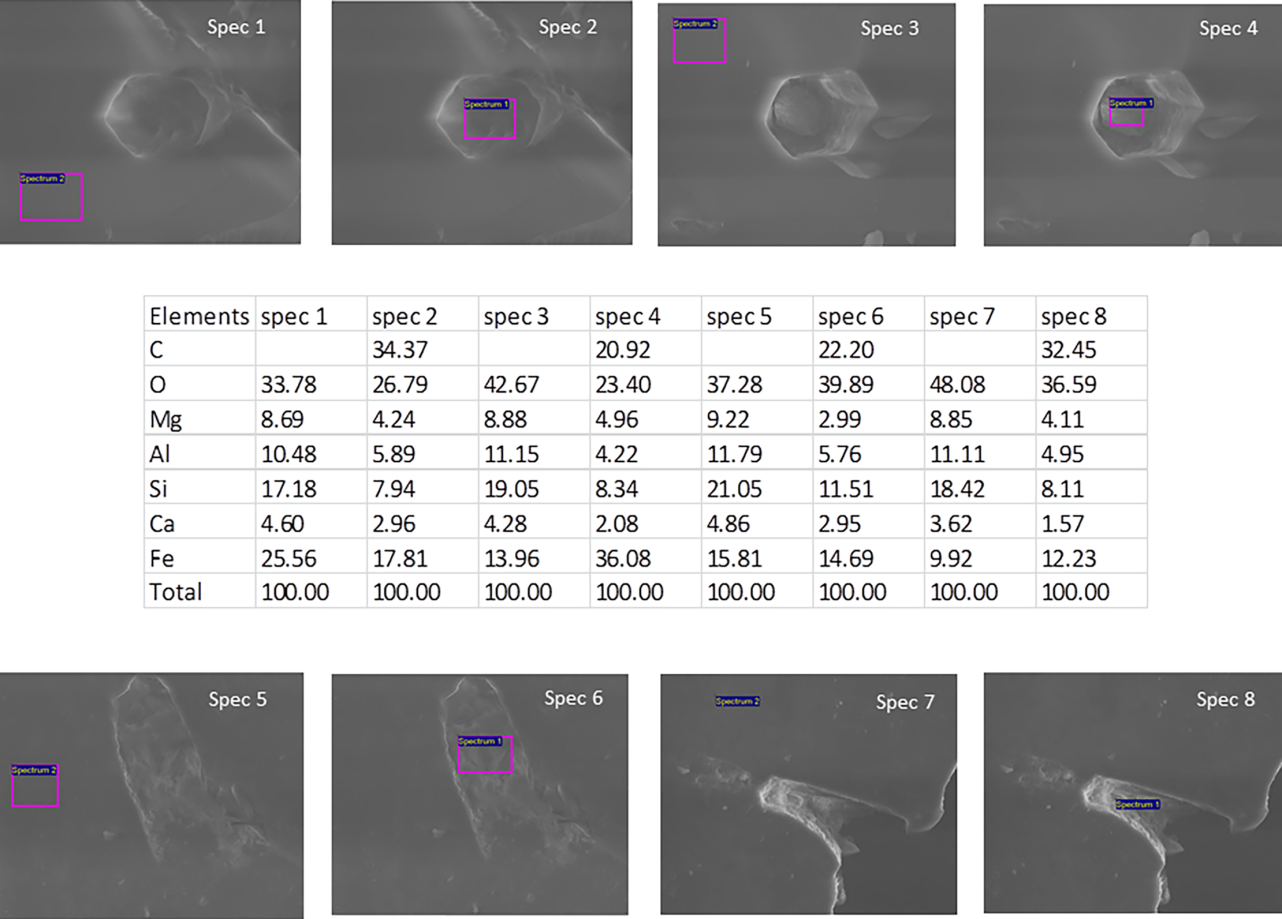
EDS data of the tunnel content. Spec 1–4: CT garnets, spec 5–6: KW garnets and spec 7–8: BR garnets. All measurements have been done on freshly cracked garnets. Thus, the analysed tunnels were exposed only seconds before being introduced to the vacuum chamber of the ESEM system. All tunnel analyses are presented with data from reference spectrum of the garnet.

Most tunnels are filled with a poorly crystalline phase that consists of O, Fe, C, Mg, Al, Si and Ca, according to EDS analyses (Fig 5). This corresponds to some type of clay phase that could represent the weathering products of the garnets. The C content of this phase varies from ~10 to ~40 wt%. The atomic ratios of C to Mg + Fe + Ca are greater than unity, which suggests the presence of carbon in addition to that bound to a carbonate phase.

Some tunnels contain filamentous structures with a diameter of ~5–15 μm and lengths of at least a few hundred micrometers (Fig 4D–4F). The filaments are curvi linear and usually of nearly the same diameter as the tunnel they exist in. The filament diameters are coherent throughout their lengths and the filaments surfaces are smooth and regular. They are reddish in optical microscopy and sometimes covered by anhedral precipitates (Fig 4D). No correlation between tunnel morphology and filament occurrence could be observed.

**Fig 6:**
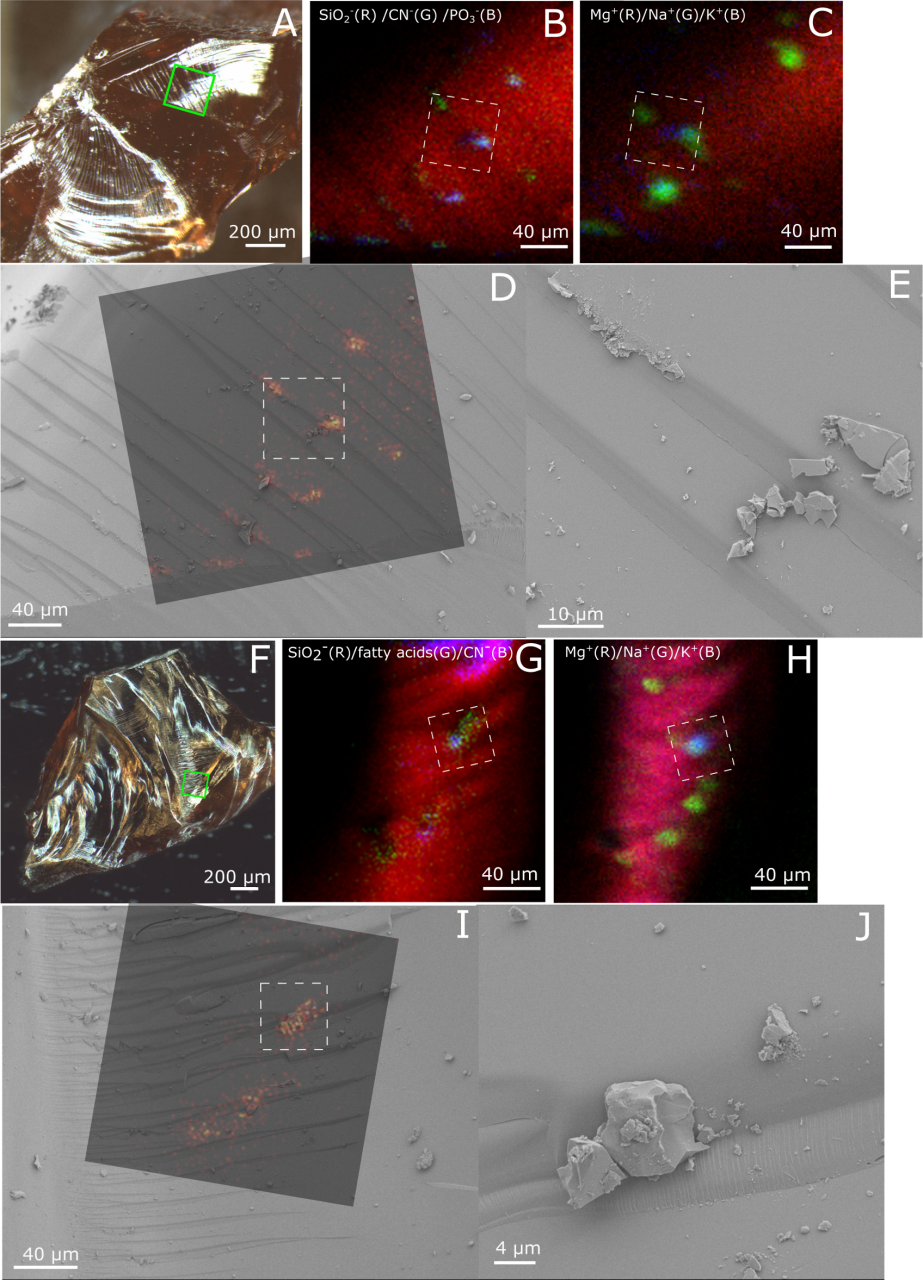
ToF-SIMS and SEM images of two tunnel-containing regions in two different garnets; A-E: CT pyropes, and F-J: KW pyropes. A) Micrograph of first garnet. Green square indicates area of ToF-SIMS analysis. B) ToF-SIMS negative ion image overlay of SiO_2_^-^ (red), CN^-^ (green) and PO_3_^-^ (blue). C) ToF-SIMS positive ion image overlay of Mg^+^ (red), Na^+^ (green) and K^+^ (blue). D) ToF-SIMS ion image of CN^-^ (green in B) overlain a SEM image of the same area. White square in B-D) indicate area of SEM image close-up shown in E). F) Micrograph of second garnet. Green square indicates area of ToF-SIMS analysis. G) ToF-SIMS negative ion image overlay of SiO_2_^-^ (red), and fatty acids (green, added m/z 241, 255, 269 and 283) and CN^-^ (blue). H) ToF-SIMS positive ion image overlay of Mg^+^ (red), Na^+^ (green) and K^+^ (blue). I) ToF-SIMS ion image of fatty acids (green in B) overlaid a SEM image of the same area. White square in G-I indicate SEM image close-up shown in J).

**Fig 7:**
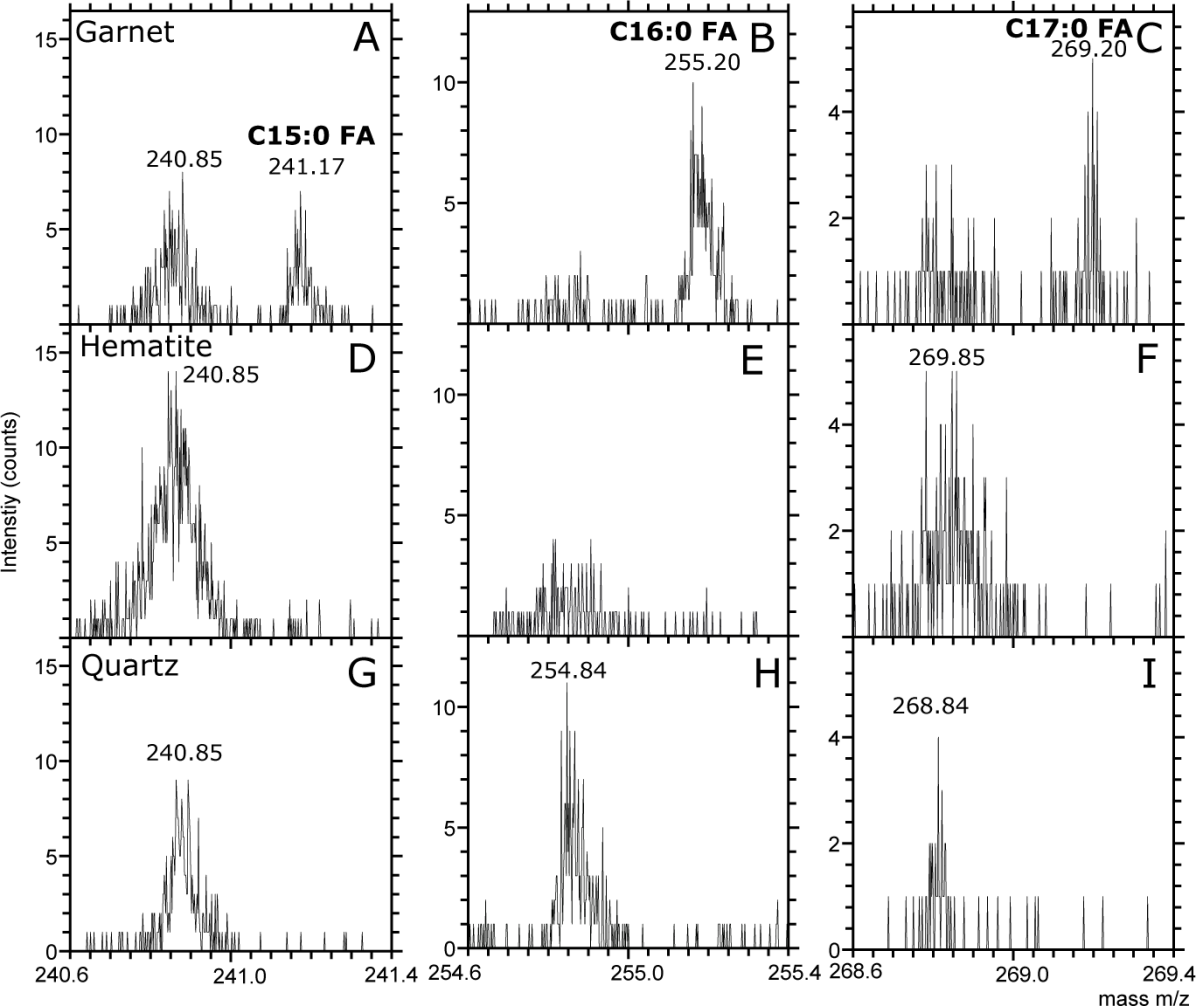
Negative ToF-SIMS spectra of freshly fractured surfaces of three different minerals: A, B, C) garnet, D, E, F) hematite, and G, H, I) quartz. Fatty acid peaks are found in spectrum of the garnet at m/z 241.17 (A, D, G), m/z 255.20 (B, E, H), and m/z 269.20 (C, F, I). All spectra were performed for 200s on 200x200 μm^2^.

ToF-SIMS analyses of fresh fracture surfaces of garnets from both KW and CT show a high organic content localized to newly exposed tunnels. The ToF-SIMS ion images show that peaks that can be assigned to CN^-^ and CNO^-^ (m/z 26.00 and 42.00) are localized to individual tunnels (Fig 6). In addition, 2 out of 5 analysed tunnel regions show signals of saturated and unsaturated fatty acids such as C_15:0_ (m/z 241.17), C_16:0_ (m/z 255.20), C_16:1_ (m/z 253.18), C_17:0_ (m/z 269.20) and C_18:0_ (m/z 283.22) fatty acids (Fig 6G) [29,30]. Co-localized to areas of high organic signal in the ion images are signals of PO_2_^-^, PO_3_^-^, Na^+^ and K^+^. When ion images of CN^-^ and fatty acids are overlaid over SEM images of the same areas, it is seen that the organic signals co-localize with tunnel structures exposed by the fracturing of the garnets. As negative controls, analyses were done on hematite and quartz grains from the same sediments as the garnets were obtained from (Fig 7). These minerals were, just as the garnets, split to expose fresh fracture surfaces and mounted on tape. They were then analysed on both the outer mineral surfaces and fresh fracture surfaces with ToF-SIMS. Spectra of fresh fracture surfaces of hematite and quartz lack fatty acid signals while spectra of the outer mineral surfaces show traces of fatty acid signals at m/z 241.17, 255.20 and 269.20. However, the fatty acid signal is always less intense in spectra collected on hematite and quartz surfaces compared to the spectra of the garnet tunnels.

## Discussion

### Endolithic presence in the garnets

The organic content of the garnet interior detected by ToF-SIMS and the complex nature of these organic molecules indicate microbial presence within the tunnel system of the garnets. C_16_ and C_18_ fatty acids are part of membrane lipids found in numerous organisms, including bacteria and eukaryotes. The presence of intact fatty acids would indicate a fairly recent deposition, though there are cases of long-time preservation of fatty acids [31–33]. There are no strictly abiotically formed tunnels among the samples to be used as negative controls; all exposed tunnels belong to the type currently described and they all have a similar content of organic compounds. The garnet surfaces, however, were investigated without any organics detected, and reference minerals (hematite and quartz) from the sediments showed no fatty acids in the exposed interiors and slight signals from the old mineral surfaces. River sediments and soils both are environments where organic compounds are abundant, and the possibility that the organic compounds were introduced into the tunnels by fluids and later concentrated by phyllosilicates cannot be entirely ruled out. However, the lack of organic compounds on the garnet surfaces, the relative complex nature of the organic compounds and their abundance throughout the tunnels indicate that they likely represent remnants of endolithic communities once living in the network of tunnels in the garnets.

The filamentous structures observed in the tunnels can either be explained as 1) a mineral phase produced by the weathering of the garnets, or 2) a biological remnant. The filamentous structures follow the varying morphology of the tunnels ranging from relative straight to curvi linear and are circular in diameter rather than flakey like phyllosilicate minerals. The clay-like compositions of the tunnel infillings including the filaments are similar to the composition of the garnets, and it is likely that they represent secondary clay-like products from weathering of the garnets, either chemical or biological in nature. Their morphology, size and occurrence are similar to those of known microbiological life [34]. Their extensive length and shifting between straight to curvi linear is found among most types of filamentous microorganisms, from prokaryotes to eukaryotes. The length of a few hundred micrometers corresponds to long filamentous bacteria like cyanobacteria and actinobacteria, but also to fungal hyphae or filamentous algae. However, there are no preserved morphological characteristics that are indicative of one type of microorganism over another. The predominant network architecture, including frequent branching and even anastomosis, is similar to mycelium-like networks seen among fungi and actinobacteria. However, the diameter of the observed filaments exceeds the diameter of actinobacteria (0.5–1.5 μm in diameter) [35,36] but corresponds more to fungal hyphae (2–27 μm in diameter) [37], and anastomoses are rare or absent among actinobacteria but common among fungal hyphae [35,38]. The filaments in the tunnels at the other end of the morphological range consisting of straight tunnels in parallel rows could reflect endoliths directed to a common energy source like photoautotrophs towards the sun. In the shallow river sediments this could be an explanation but not at depth in the soils, and the straight type is found in both environments. Besides, morphological comparisons of microbial communities in an open medium with those in substrate galleries are not fully applicable and should be treated with caution.

While the presence, or past presence, of microorganisms in the tunnels is strongly indicated by the ToF-SIMS data, a possible microbial involvement in the production of the tunnels is not as easy to determine.

### Secondary tunnel formation in garnets

All tunnels in the current investigation are rooted at the grain surfaces, which indicates that they are the result of secondary weathering processes. The mineral grains have been subject to an external agent of chemical, physical or biological nature after their formation. This interpretation is supported by tunnels that originate at relatively fresh surfaces, exposed by chemical or physical weathering. Comparison of the samples from OL and CT further support the interpretation. Since the unweathered garnets in the host rock (OL) are featureless, lacking the characteristic tunnels found in eroded garnets downstream (CT), it is evident that the tunnel structures are formed during the downstream transport in the river system. The river is a low-energy system with calm waters, which suggests that mechanical force is not likely to be responsible for the tunnel production, even if that were possible in a high-energy system. It is more probable that the tunnels have been formed in the river sediments by some other type of weathering process. The samples from KW, BR, NB have been weathered in situ, and thus by chemical rather than mechanical forces.

These observations exclude original mineral inclusions as an explanation of the tunnels. For instance, pyrope and almandine garnets are known to contain needle-like inclusions of mainly rutile. However, those are normally uniformly distributed following garnetohedral faces within crystals [39], and are not curvi-linear and rooted at surfaces as in the present samples. Another type of structure of magmatic origin with a fibrous appearance is so called horsetail-inclusions which occur in the andradite-variety “demantoid”. Those usually consist of chrysotile fibres and radiate in all directions from a central chromite grain and, thus, are not rooted at the surface. Another strong argument against tunnels being caused by some unusual fibrous mineral inclusions is the fact that they occur in a wide range of samples of different composition and occurrences, and are thus not restricted to specific geological circumstances.

Chemical weathering of garnets can produce surficial etch-pits and erosion patterns with a polygonal appearance, but tunnel structures have not been reported before [40]. Chemical weathering of garnets in lateritic environments or saprolites has been shown to produce shallow polygonal etch pits of varying diameter, but no deep galleries. Weathering of almandine usually results in a secondary surface layer of weathering products like goethite, kaolinite or pyrolusite that cover the entire mineral surface [41]. Beneath the surface layers there are usually polygonal etch pits being observed.

Complex tunnel structures, as in the current study, are not likely to be formed exclusively by chemical dissolution but need the involvement of an agent that controls the direction [6]. Abiotically produced tunnels can be ambient inclusion trails (AITs), fluid inclusion trails or radiation damage trails. The last two structures can be dismissed for morphological reasons and because of how they appear in the mineral grains. Neither type is necessarily rooted at the mineral surface, as all tunnels in the current study are, but both can originate in the middle of grains and crystals. Fluid-inclusion trails are straight, not curvi linear, and usually associated with strings or clouds of fluid inclusions yet to be exploited by tunnelling [7]. Radiation damage trails should occur with random orientations in the vicinity of a source of radioactive particles, and may intersect one another like fission trails. Radiation damage trails usually originate in the interior of a grain or crystal.

AITs are usually described as the result of mineral grains having been propelled through a lithified substrate leaving a tubular microcavity behind [42]. AITs are characteristic in appearance with longitudinal striae, polygonal cross-section, uniform diameter, and sometimes a terminal grain [7,13]. Some features in the current tunnels are consistent with AIT formation, such as the polygonal cross section and the smaller diameter of side branches compared to the original tunnel. In AITs the diameter of the side branches is less than that of the original tunnels because of splitting of the propelled grain. However, a number of features speak against AIT formation of the tunnels, such as lack of terminal grains, lack of longitudinal striae, lack of consistency in diameter throughout the tunnels, consistent orientation of the polygonal cross sections, lack of polygonal cross section throughout the tunnels, and lack of consistency at branching, where the side branches sometimes have the same diameter as the original tunnel and sometimes not. Especially the diameter of AITs needs to be consistent since they reflect the diameter of the grain, and not varying in diameter as the tunnels in the current garnets. Tapering of a tunnel is not consistent with AIT formation.

There are also aspects of mineral hardness and geological context that speak against an AIT interpretation. AITs are formed by a mineral grain, usually metal-rich in composition, which is propelled through a substrate with a lower hardness on Mohs`scale compared to the millstone. For example, most AITs are formed by pyrite or in some cases magnetite crystals propelled through carbonate or phosphate [13]. In those cases the substrate has a hardness of 4–4.5 while the propelled material have a hardness around 6.5–7, thus a difference in hardness of about 2 units on the Mohs`scale. A corresponding hardness ratio for the current garnets (H_pyrope_ = 7.5), would require a mineral millstone with a hardness of 9 or above to form AITs. Possible candidates would be corundum (H_corondum_ = 9, including the varieties sapphire and ruby) or diamond (H_diamaond_ = 10). Such minerals are absent in the river sediments and extremely rare in the residual soils [19–24,39]. Besides, considering the number of tunnels in one single garnet (sometimes more than 100), an excess of such mineral grains would have been needed in these environments to form the garnet tunnels. That is simply not the case in any of the examined localities [21–26,43].

There are also examples where a pyrite grain has been propelled through a chert-like substrate [10]. In such a case we have a penetrated substrate and a propelled grain of about the same hardness. However, the process behind the AIT formation is pressure solution initiated by gas evolution from organic material attached to the pyrite millstone [44]. As the original sediment that preceded the chert was subject to metamorphosis, a pressure was build up by heating of the organic material resulting in the pyrite grain being pushed through the sediment/partly lithified chert. Thus, initially, the pyrite was not propelled through a substrate of the same hardness but in an unlithified or partly lithified sedimentary rock. Later on as the lithification proceeded the progress of the pyrite was forced by metamorphosis of prehnite-pumpellyite facies [13], which the samples of our current study not have been subject to [21–26,43]. The tunnels in the current garnets have been produced in surficial river sediments and in weathered soils, respectively, and thus not been subject to pressures and temperatures that could have heated and decomposed organic matter to the degree of pushing a crystal through the garnet. Considering the above arguments, including both the morphological features and the geological aspects, an AIT interpretation of the tunnels is excluded.

The complexity of the networks with anastomoses between branches further rules out AITs. Anastomosis is in fact exclusively a biological feature but anastomosing tunnels produced by endolithic microorganisms have not yet been reported. Thus, even though the tunnels, at least partly, might look non-biogenic at first glance there is no conceivable non-biological mechanism that can explain the formation of them.

### Influence of crystallography

The hexagonal cross section of the tunnels, usually the marginal portions, is probably due to the cubic crystallinity of garnets. Chemical weathering of garnets would be expected to reflect the crystallinity and produce hexagonal etch marks according to the crystal structure. However, the propagation of the network of curvi linear, branching and anastomosing tunnels appears to be independent of crystallography. Even though their propagation is not random, considering anastomosis and relation to mineral surfaces, they occur independently of the mineral crystallinity. The exception is the linear arrangement of straight tunnels with a coordinated curvature. Such mutual kink of the tunnels suggests influence from the crystal medium. Many garnets have growth zonations of dodecahedral form, parallel to the mineral surface. Such zonation zones are weakness planes in the crystals that any type of weathering, mechanical, chemical or biological, would take advantage of. Thus, despite the type of weathering agent responsible for the coordinated curvatures, it is probable that it has followed mineral weaknesses, and in these specific cases they coincide with growth zonations of dodecahedral form.

### Biological origin of the tunnels

A possible biological explanation for the production of the tunnels needs careful and critical consideration. The polygonal cross section, the variations in diameter and sometimes large diameter (up to ~40 μm), and occasional straight appearance are features that do not correspond to known inferred biologically produced tunnel structures [7,13]. However, that fact that all tunnels are rooted at the mineral surfaces supports the idea of an external responsible agent, and life is one alternative. The tapering of tunnels from wide, polygonal etch-like marks at the surfaces to thinner tube-like structures, circular in diameter, further into the minerals suggests a later biological influence. Branching is common among filamentous microorganisms but can arise abiotically in, for instance, AITs, so branching is not a conclusive criterion.

In support of a biological formation is also the presence of filamentous structures most likely representing filamentous bacteria or fungal hyphae, and the presence of fatty acids in the tunnels detected by the ToF-SIMS analyses. Fungal hyphae are known to excrete organic acids as well as siderophores and other chelators at their hyphal tips that can mediate mineral dissolution [14,15]. Fungal hyphae can also function as a transport system in which dissolved nutrients and elements can be removed from the interior of the tunnels and prevent clogging of the bored tunnel by byproducts of the mineral dissolution and subsequent biomineralisation [6,14,15].

To evaluate the microbial involvement, the tunnels should be tested against biogenicity criteria for trace fossils. Biogenicity criteria for trace fossils are usually formulated with reference to the substrate and are most commonly boiled down to three main criteria: 1) is the geological context compatible with life and can the syngenicity of the biological remains be demonstrated, 2) evidence of biogenic morphology and behaviour; and 3) geochemical evidence for biological processing [7,13,45–47]. The following is a test to address these criteria: 1) The environment in which the formation of the tunnels took place has been deduced to river sediments for the CT samples and residual soils from weathering of basalts for the KW, NB, and BR samples, which both represent environments in which microorganisms flourish. The formation of the tunnels has been shown to occur at a late stage in the weathering of the garnets, and the morphological and the biomarker evidence have been proven indigenous to the tunnel structures. Thus, criterion 1 is fulfilled. 2) The garnet tunnels show morphological features that can be interpreted as both biological and abiotic. However, as discussed in the previous section, the majority of the morphological characteristics speak against an abiotic interpretation and are in favour of a biological interpretation. Thus, the second criterion supports a biological explanation over an abiotic. 3) The ToF-SIMS data are indicative of a past or relative recent occurrence of microorganisms in the tunnels, as is the presence of filamentous structures similar to filamentous microorganisms. However, neither the chemical data nor the filaments are evidence for microbial boring, but only for an endolithic presence. The criterion is fulfilled, but cannot be used as conclusive evidence for microbial boring.

Eventually, biogenicity criteria are in favour of a biological explanation of the tunnel structures in the garnets even though an abiotic influence cannot be fully dismissed. There are morphological features that suggest combined abiotic and biological processes. The transition from polygonal entrance pits at the mineral surfaces to more circular and tapering tunnels further into the minerals suggests that the tunnels were initiated by abiotic processes or a combination of abiotic and biological processes, which further into the mineral shift to predominantly biological processes. The presence of organic matter and bio-related elements in association with AITs has been suggested to be of biological origin and explain the progress of the pyrite grain by pressure release as a result of decomposition of organic matter [13]. Lepot *et al*. [48] further showed that decomposition of organic matter was the driving force behind AIT formation in agate by garnet crystals. The resulting tubes were rich in carbonaceous material and similar in appearance to microfossils and, thus, easy to mistake for true fossilized microorganisms. Ménez *et al*. [18] showed high amounts of complex organic molecules like lipids, proteins and nucleic acids associated with hydrogarnets in subseafloor serpentinized peridotites. The organic content was indicative of past microbial activity, and the garnets showed similar polygonal etch marks as in the current study. Many of the etch marks were filled with a C-Mg-Si gel interpreted as the remnants of microbial communities.

A similar microbially driven process is easy to assume for the early stages of garnet etching in the current study. The garnets were probably subject to various chemical and/or biologically driven processes in the river sediments and soils that dissolved the garnets surficially and left polygonal etch marks. After a first chemical/biological weathering stage a second stage was initiated by the colonization of an organism equipped with hyphae or equivalent structures that were able to chemically dissolve and penetrate the mineral, and also transport the weathering products out of the produced tunnels while carbon for further growth was transported into the tunnel networks. In that process the complex networks that we see today were formed. The morphology of many tunnels supports this interpretation, since they have a wide polygonal entrance at the mineral surface but taper off further in and lose their polygonal shape in cross section.

Fungi are known from various subaerial and subsurface environments to dissolve minerals, metals and building stones usually with the result of anhedral rock decay but sometimes by tunnels [14,15,49,50]. This occurs both in symbiotic relationships like lichens but also as free-living forms [49,50]. Mycorrhizal fungi, for instance, bore into soil minerals to mobilize and transport nutrients to their symbiotic plant, leaving tunnels behind [4,5]. In subseafloor igneous crust fungal hyphae have been shown to be responsible for abundant tunnelling in secondary mineralizations like carbonates and zeolites [10,11], and it has been suggested that tunnels in subseafloor glass is produced by fungal hyphae [6].

Biomechanical weathering is normally performed by hyphal penetration along crystal planes, cleavages or other crystal weaknesses but seldom results in tunnels [49,51]. Fungal tunnelling are usually dependent on chemical weathering usually through the production of organic acids and chelators like siderophores to complex metal ions [50], but also through redoxolysis and carbonic acid attack formed as a result of respiratory CO_2_ production [52]. It has however, been shown that fungal tunnelling in minerals is performed by a combination of both chemical and mechanical influence. The hyphal growth at the fungi-mineral interface subjects a mechanical force that is indispensable for the destruction of the crystal lattice and further penetration into the mineral [53]. Growth of fungal hyphae in minerals has also been shown to be directed towards nutrients and reflects element distribution within a mineral. In feldspars, fungal tunneling follow nutrient gradients [54], which could explain the palisade appearance of some of the straight tunnels.

A fully satisfying explanation for the anastomosing behaviour of the tunnel systems cannot be offered. The only known natural processes that can form anastomoses in three dimensions are biological, but microscopic trace fossils with such features have so far not been reported. Abiotically formed dendritic structures can form anastomosing patterns along a flat plane by random interconnectedness between branches by, for instance crystal growth, but not true anastomosis in three dimensions. There is also a significant difference between anastomosis in a mycelium within an open medium like air or water, and an anastomosing network of tunnels formed by an organism in a hard substrate. Common tropism, directed growth as a response to environmental stimulus, known among plants but also fungi [55] is only known from open media and cannot explain the apparent organized direction of the tunnels that results in anastomosis. Formation of anastomosing tunnels by biology would require some type of communication between separated organisms or at least organismal parts such as different hyphae of a fungal mycelium within a substrate. Such communication could be chemically controlled by fungi excreting molecules at the hyphal tip [56]. Another mode of communication in a transparent substrate could be light. Natural bioluminescence is known among fungi to attract invertebrates for spore dispersal or as warning signals to repulse fungivores, but not for communicative purposes [57,58]. Without supporting observations among live species in controlled laboratory experiments fungal communication within a substrate is so far hypothetical.

### Reasons for biological tunnelling

Reasons for biological boring are usually acquisition of habitable space or trophy. The Fe component in pyropes and almandines is Fe^2+^, which can be oxidized by Fe-oxidizing microorganisms. Mössbauer analyses of the current garnets show that Fe^2+^ is the main Fe constituent (Fe^2+^/ƩFe = 0.92–0.99) [20]. In weathered and oxidized soils and river sediments where garnets are among the only phases left containing Fe^2+^ they would be an obvious target for Fe-oxidizing microorganisms. In the soil samples it could also be fungal hyphae of mycorrhiza that is dissolving Fe^2+^ and transport it to plants. The diameters of the filaments found in the tunnels match both known iron oxidizing bacteria [34] and fungal hyphae but the length and branching as well as anastomosing behaviour exclude bacteria but matches fungal hyphae [14,15]. Lack of Gallionella-like spiral morphologies are also in disagreement with a bacteria interpretation [34]. Whether or not the tunnels are biologically produced, garnets in river sediments and weathered/oxidized soils with intricate tunnel systems represent a previously unknown niche for endolithic microorganisms.
